# Envisat MERIS and Sentinel-3 OLCI satellite lake biophysical water quality flag dataset for the contiguous United States

**DOI:** 10.1016/j.dib.2019.104826

**Published:** 2019-11-16

**Authors:** Erin A. Urquhart, Blake A. Schaeffer

**Affiliations:** a Oak Ridge Institute for Science and Engineering (ORISE), US Environmental Protection Agency, Durham, NC 27709, USA; b US Environmental Protection Agency, Office of Research and Development, National Exposure Effects Laboratory, Durham, NC 27709, USA

**Keywords:** Sentinel-3, OLCI, Envisat, MERIS, Remote sensing, Biophysical water quality, Lakes, Satellite, QA, Quality Assurance, CONUS, Contiguous United States, SRTM, Shuttle Radar Topography Mission, SWBD, SRTM Waterbody Data, DN, Digital Number, NASA, National Aeronautical Space Administration, OBPG, Ocean Biology Processing Group, ESA, European Space Agency, MERIS, Medium Resolution Imaging Spectroradiometer, OLCI, Ocean Land Colour Instrument, SEADAS, SeaWIFS Data Analysis, NHD, National Hydrography Dataset, NLA, National Lakes Assessment

## Abstract

Monitoring lake biophysical water quality is a global challenge. Satellite remote sensing offers a technology for continuous water quality information in data poor regions throughout the United States. Quality assurance flag data are provided for the presence of snow/ice, land-adjacency, and unresolvable waterbodies supporting water quality derived measures from Envisat MEdium Resolution Imaging Spectrometer and Sentinel-3 Ocean and Land Colour Instrument for the continental United States. In addition, an updated Waterbody Data mask that contains valid waterbody and coastal ocean delineation is provided. The quality assurance flag datasets can benefit the scientific community in processing lake water quality throughout the contiguous United States by addressing errors from snow/ice, land adjacency, and land masking. The dataset presented here will be used in the development of national scale metrics for derived biophysical water quality in the US.

**Table:** Specifications Table

Subject	Environmental Sciences
Specific subject area	Remote sensing, hydrology
Type of data	Satellite quality assurance flags Polygon Shapefiles
How data were acquired	**Satellite data**: European Space Agency (ESA) Envisat Medium Resolution Imaging Spectroradiometer (MERIS); ESA Sentinel-3 Ocean Land Colour Imager (OLCI); **National Snow Ice Data Center**: 4km snow coverage raster maps; **Waterbody Data**: Research Environments (MEaSUREs) Shuttle Radar Topography Mission (SRTM3.0) V3
Data format	Raw GeoTiff Netcdf Spatial Polygon Shapefile
Parameters for data collection	Data were collected from Envisat MERIS and Sentinel-3 OLCI imagery within the contiguous United States. Quality assurance flags were created using the raw satellite data for inland lakes. Coastal and inland waterbody data were collected and updated using the SRTM 3.0 dataset.
Description of data collection	Satellite quality assurance flags were compiled, filtered, and processed to produce masking maps of for biophysical water quality monitoring in the contiguous United States lakes.
Data source location	Contiguous United States of America, i.e., (north-south) from 49° 22′ 48″ N to 25° 50′ 24″ N, and (east-west) from 66° 57′ 0″ W to 124° 40′ 12″ W.
Data accessibility	US EPA Environmental Dataset Gateway. https://doi.org/10.23719/1503160

**Table:** 

**Value of the Data** • This data provides snow/ice, land-adjacent pixel, and unresolvable waterbody quality assurance flags for inland lakes and reservoirs across the contiguous United States. • The quality assurance flag datasets benefit the scientific community in processing lake water quality throughout the contiguous United States by addressing errors from snow/ice, land adjacency, and land masking. • The quality assurance flags can be used to develop national-scale metrics for lake biophysical water quality parameters. • The dataset is essential in inland harmful algal bloom monitoring particularly in US northern latitude regions as it contains new snow and ice cover flags and filtering. • The updated Version 4.0 Shuttle Radar Topography Mission dataset provides corrected Rhode Island and Massachusetts inland waterbody data and corrected coastline boundary for the United States.

## 1 Data

**Fig. 1:**
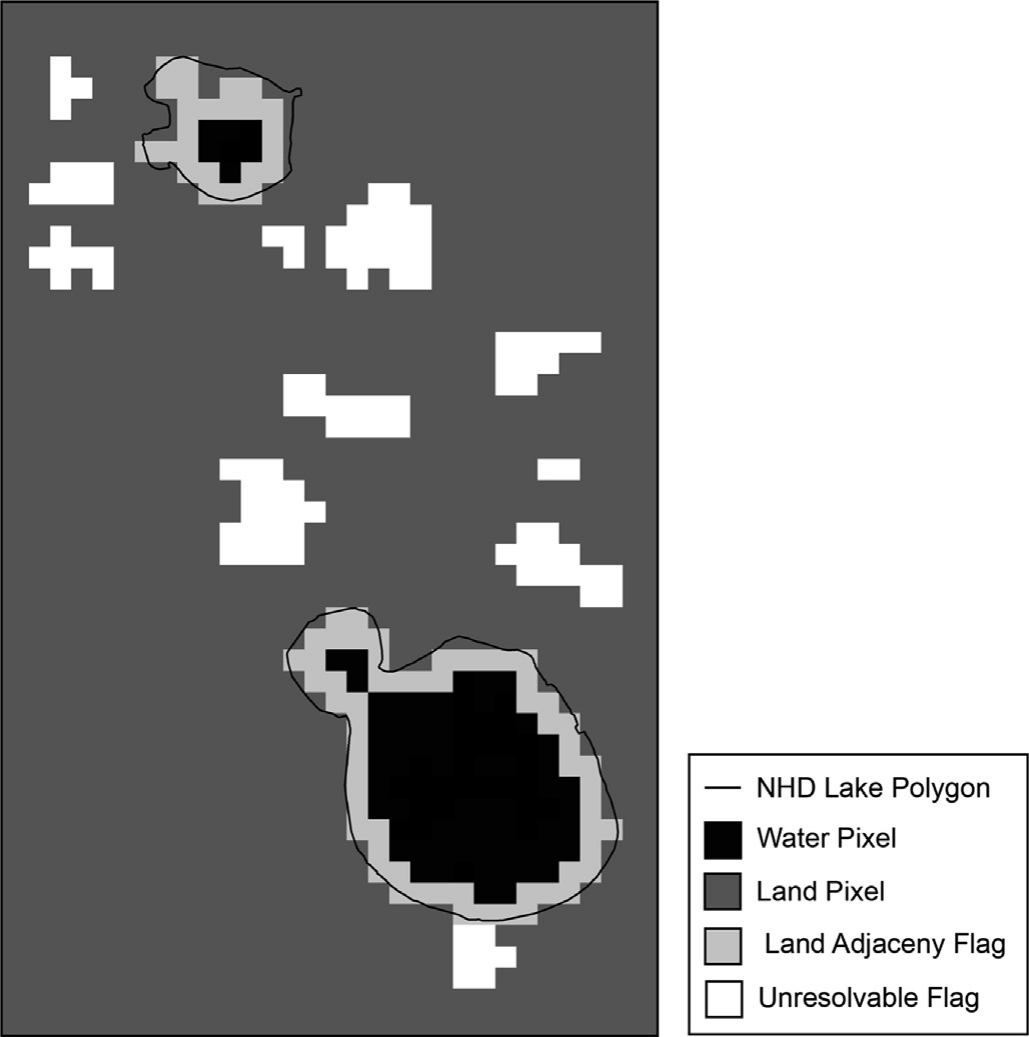
An example of QA flags for a subset of fictitious lakes. Black indicates a water pixel; dark grey is a SRTM land pixel; light grey is a land-adjacency QA flag pixel; and white identifies an unresolvable water body pixel not within a valid NHD Lake Polygon.

The dataset contains the added pixel quality assurance (QA) flags that help ensure validity of satellite-derived biophysical water quality estimates in freshwater lakes and reservoirs. This work builds upon a study by et al. [1] where inland lake and waterbody Envisat MERIS satellite data was processed and flagged for California, Ohio, and Florida. Added pixel QA flags include land-adjacent pixels, unresolvable waterbody pixels, and snow/ice pixel mask at 300 m spatial resolution. Future work may provide a similar set of QA flags and snow/ice datasets for higher resolution sensors in the 10 m–60 m spatial resolution range such as the Copernicus Sentinel-2 mission. Any water pixel adjacent to land is flagged to caution potential for mixed land-water pixels and land adjacency effects. A weekly QA flag mask is provided for snow/ice presence over lakes. The unresolvable QA flag mask contains inland waterbodies smaller than 27 ha and/or with less than three 300 m resolvable satellite pixels. Fig. 1 illustrates a simple schematic of example waterbodies with Land Adjacency QA and Unresolvable Waterbody QA flags applied to a generic Sentinel-3 Ocean and Land Colour Instrument (OLCI) satellite image.

The satellite data for this article were obtained from the National Aeronautical Space Administration (NASA) Ocean Biology Processing Group (OBPG) [2]. The data contains QA flags for Envisat and Copernicus Sentinel-3 data provided by the European Space Agency (ESA). Full resolution (300 m), weekly satellite imagery from Medium Resolution Imaging Spectroradiometer (MERIS) and OLCI were downloaded for the contiguous United States (CONUS). Level 3 MERIS and OLCI data were processed by NASA OBPG using their standard satellite ocean color software package SeaWIFS Data Analysis System (SeaDAS), the Shuttle Radar Topography Mission (SRTM) Waterbody Data (SWBD), and a transformation to Albers Equal Area with an area-weighted interpolation to match the projections of the National Hydrography Dataset (NHD) High Resolution Dataset [3]. Quality assurance flagging and masking of cloud cover, cloud shadow, and glint are already applied by the NASA SeaDAS Level 3 processing [4]. Level 3 data are temporally and spatially aggregated and projected onto an equal area grid with standard bin sizes over a specified time period.

**Fig. 2:**
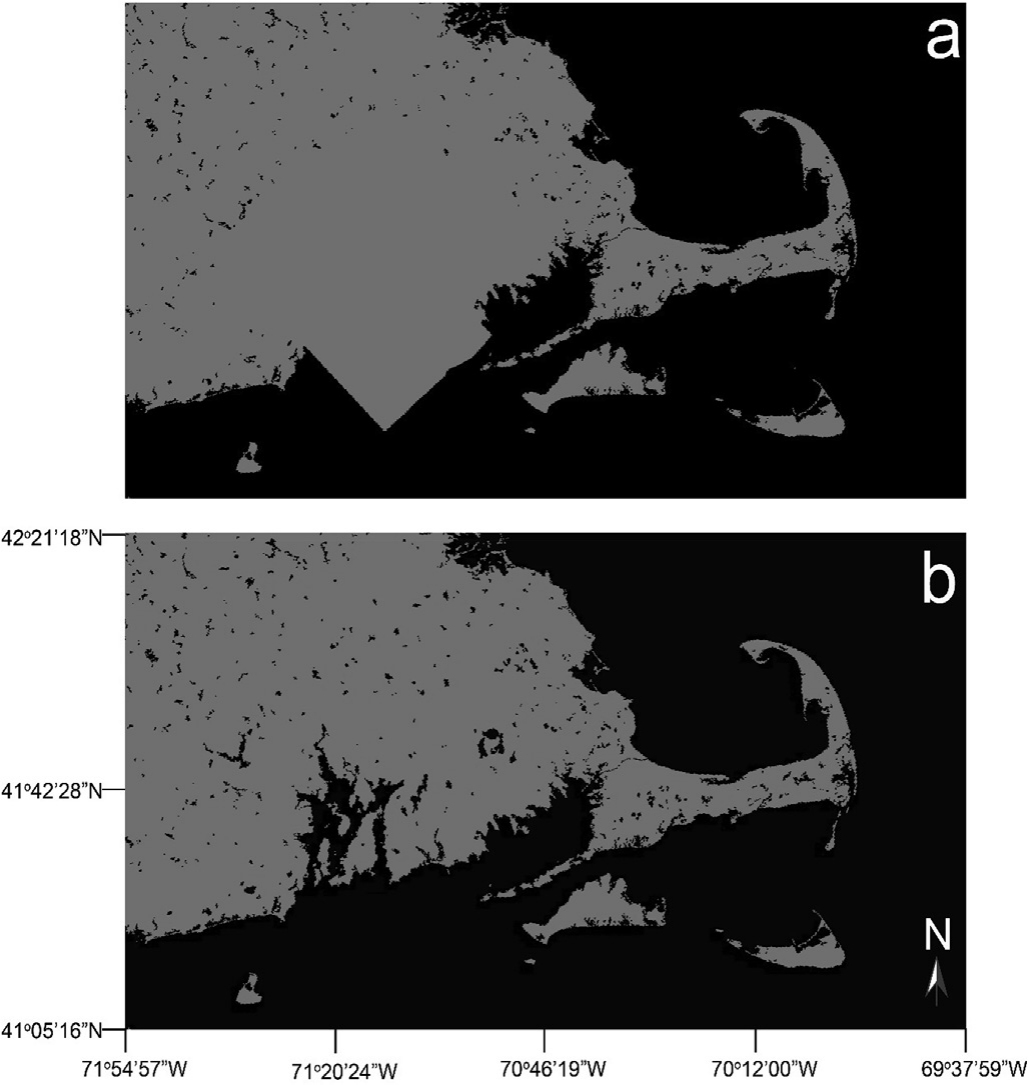
Version 3.0 (a) and Version 4.0 (b) of the SRTM Waterbody Data. Grey color indicates land pixels, black color is water pixels. Illustrates the coastal boundary and inland waterbody corrections in Rhode Island and Massachusetts in Version 3.0 versus Version 4.0.

An updated version of SRTM SWBD is provided to fix a land-waterbody mask error identified in Rhode Island and Massachusetts. The Research Environments MEaSUREs SRTM, used in the NASA data processing, includes the SWBD shapefiles (∼30 m) product. Version 3.0 of the SRTM contains the SWBD vectorized coastline available in both shapefile and rasterized formats [5]. Version 4.0 of the SRTM fixes the land-waterbody mask error identified in Rhode Island and Massachusetts. Waterbody Data updates are illustrated in Fig. 2b with land pixels colored grey, and water pixels colored black. Version 4.0 of the SRTM has been adopted into the NASA processing of the MERIS and OLCI QA flags described above.

## 2 Experimental design, materials, and methods

### 2.1 SRTM version 4.0 Waterbody Data

Visual inspection of NASA processed satellite data identified errors in the SRTM SWBD 3.0. Specifically, inaccurate coastline was identified in Rhode Island and Massachusetts (Fig. 2a). A USA polygon boundary (USA States 1:3 m; ESRI ArcGIS Online) was used to update the SRTM SWBD coastline in interest. Nearby lakes and reservoirs were also mistakenly excluded from the original SRTM SWBD, showing land pixel values in place of real waterbody locations. Correct lake water pixels were identified and added to the SRTM SWBD using the NHD high-resolution lake shapefiles for waterbodies (1:24,000 scale or better) within Rhode Island and Massachusetts. NHD lakes containing/intesecting at least three satellite raster pixel centroids were added. Additionally, per the request of the state of Ohio, Doutt Reservoir in Ohio was added to the dataset as it was incorrectly identified as land pixels values instead of water pixels in SRTM 3.0.

### 2.2 Land adjacency QA flag and unresolvable waterbody QA flag

Archived full-resolution (300 m at nadir), MERIS and OLCI data were obtained over the contiguous United States. A spatial mosaic composed of 54 individual satellite scenes across CONUS was generated resulting in one raster GeoTiff file for the country. A United States boundary polygon shapefile (USA States 1:3 m; ESRI ArcGIS Online) was used to mask and exclude any ocean, estuary, or inlet/bay artifacts of the raw satellite data. Any water pixel directly adjacent to the SRTM land mask was add to the Land Adjacency QA flag to caution potential for mixed land-water pixels and land adjacency effects such as bottom reflectance.

Water pixels were extracted using the NHD polygon dataset for each inland lake or waterbody. All NHD features classified as lakes and reservoirs were selected using US Environmental Protection Agency's 2012 National Lakes Assessment (NLA) site evaluation guidelines [6,7]. Lakes in the NHD shapefile with a minimum of three satellite water pixels remaining after the land adjacency QA flag was applied were considered resolvable waterbodies. Waterbodies classified as intermittent, estuarine, rivers, streams, or waterbodies with a surface area <27 ha are considered “unresolvable water” and thus QA flagged based on NLA criteria.

### 2.3 Snow and ice presence

Conventional methods to distinguish between ice and water often fail due to high ice reflectance in areas with thin ice or mixed ice and water, owing to the absorption of near-infrared by water, combined with highly reflective ice (in the visible), as well as the possibility of cyanobacterial biomass formation under the ice [8,9]. Therefore, weekly satellite data were flagged for the presence of ice and snow using the Iterative Multisensor Snow and Ice Mapping System Northern Hemisphere Snow and Ice Analysis data (Version 1.0, 4 km resolution). Daily snow and ice data were obtained from the National Snow and Ice Data Center [10] then masked to the US boundary polygon shapefile. Snow and ice data values were collapsed and temporally binned into maximum weekly (7-day) composites, then converted from raster to SpatialPolygon format. The act of temporal binning helps overcome the issue of missing data [11]. A 7-day binning period represents one week, leaving 52 or 53 weeks per calendar year with the later containing one or two days. Internal holes in the ice/snow polygons were removed, leaving the polygon topology intact, to ensure pixel coverage over waterbodies smaller than 4km^2^.

**Fig. 3:**
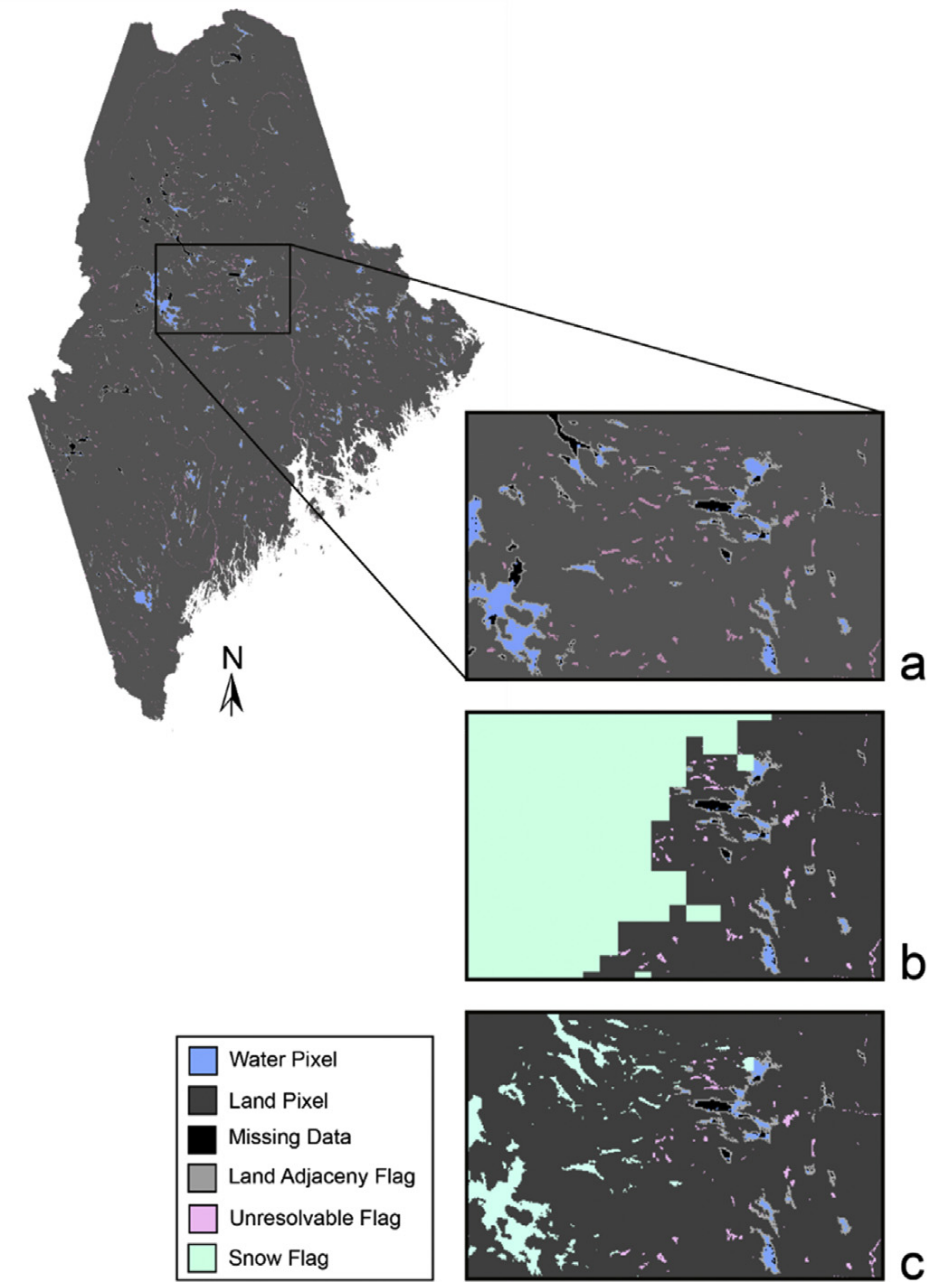
Example of all quality flags applied to a Sentinel-3 OLCI file of lakes in the state of Maine for the week of November 21, 2017. A satellite water quality data file will already have cloud and cloud shadow (a) QA flags applied as showing in black pixels. The additional QA flags of unresolvable waterbodies (pink) and land adjacent pixels (grey) are added in (a). A snow/ice SpatialPolygon mask (green) is overlaid onto the satellite data file (b) and any remaining water pixels are flagged (c).

Weekly snow/ice SpatialPolygon shapefile composites (n = 379) are provided from January 1, 2008 through April 7, 2012 and June 5, 2016 through August 1, 2019 with the following naming structure “*SYYYYDDDYYYYDDD_ice*.*shp*”. The naming convention of the weekly snow/ice shapefiles is “S” for snow/ice, “YYYY” for week start year, “DDD” for week start day, “YYYY” for week end year, “DDD” for week end day. If satellite pixel locations are within the spatial area of the snow/ice SpatialPolygon mask, it is suggested that they be omitted from further lake water quality analysis. Fig. 3 illustrates an example of a snow/ice QA flag mask applied to a Sentinel-3 OLCI file (a) of lakes in the state of Maine for the week of November 21, 2017. The snow/ice SpatialPolygon mask (green) is overlaid onto the satellite image (b) and any remaining water pixels are flagged (c).

Statistical analysis and data processing were performed in the R-statistical computing environment version 3.5.1 [12].

## Funding

This work was supported by The Environmental Modeling and Visualization Laboratory (EMVL) and High-Performance Computing (HPC) at the United States Environmental Protection Agency (EPA). This material is based upon work supported the NASA Ocean Biology and Biogeochemistry Program/Applied Sciences Program [proposal 14-SMDUNSOL14- 0001] and by US EPA, NOAA, US Geological Survey Toxic Substances Hydrology Program, and Oak Ridge Institute for Science and Technology (ORISE).
